# Massive Bleed Following Optical Internal Urethrotomy: An Unforeseen Doom Discussing the Unique Management Technique

**DOI:** 10.1089/cren.2018.0076

**Published:** 2018-11-01

**Authors:** Varinder Attri, Kalpesh Parmar, Sudheer Dewana, Gopal Sharma, Abhishek Chandna

**Affiliations:** Department of Urology, PGIMER, Chandigarh, India.

**Keywords:** optical internal urethrotomy, urethral bleed, pseudoaneurysm, angioembolization

## Abstract

***Objective:*** To demonstrate the unique management technique of ultrasound (USG)-guided compression repair for bulbourethral artery pseudoaneurysm following optical internal urethrotomy (OIU) for short segment bulbar urethral stricture.

***Methods:*** A 40-year-old man underwent day care OIU for short segment bulbar urethral stricture. The procedure was uneventful and the patient was discharged the same day. After catheter removal on day 7, the patient had massive bleed from urethra. Perineal compression stopped the bleeding; however, the patient bled again and needed blood transfusion. CT angiography revealed right bulbourethral artery pseudoaneurysm.

***Results:*** USG colour Doppler was performed to find out the exact site of pseudoaneurysm, and focused compression was given for 25 minutes. Later assessment showed no flow in pseudoaneurysm and tract was obliterated.

***Conclusion:*** Minor bleed following OIU is quite common and generally subsides spontaneously or with pressure dressing. Massive bleed is uncommon, although reported in the literature, and requires angioembolization of the feeding vessel. Before going for invasive procedure, this simple technique of USG-guided compression can be tried as it is simple, cost-effective, and highly successful in small pseudoaneurysm.

## Introduction

Urethral bleed following trauma is a well known event. It usually subsides without any complication. Minor bleeding following optical internal urethrotomy (OIU) is quite common and generally subsides spontaneously or with perineal compression. Profuse bleeding following OIU is an uncommon consequence. In refractory case, it may require invasive procedures in form of angioembolization. Ultrasound (USG)-guided compression repair is a well-defined, safe, and cost-effective therapy for peripheral pseudoaneurysm. We report the first case of urethral artery pseudoaneurysm following OIU, which was managed by USG-guided compression technique.

## Case Report

**FIG. 1.**
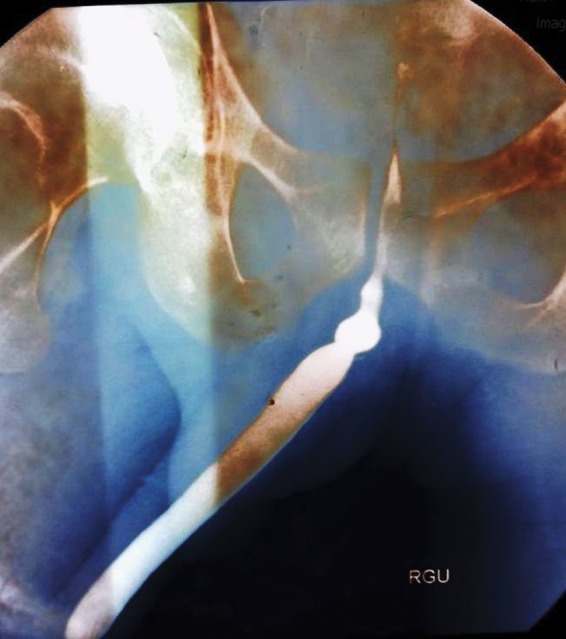
Retrograde urethrogram of a 40-year-old man showing short segment mid-bulbar urethral stricture.

**FIG. 2.**
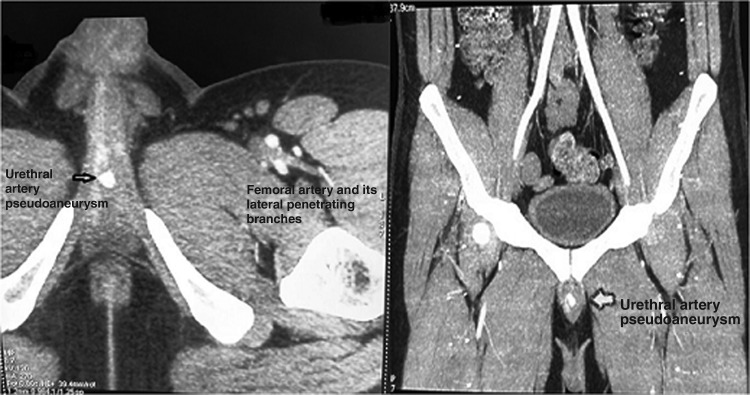
CT angiography images (axial, sagital cuts) showing pseudoaneurysm in the region of penile urethra, which was confirmed to be arising from bulbourethral artery in dynamic images.

**FIG. 3.**
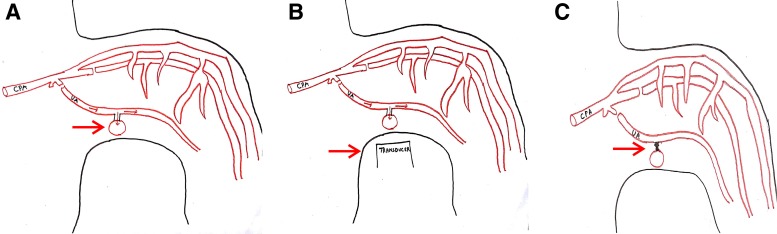
**(A)** Schematic diagram showing urethral artery pseudoaneurysm (*arrows*). **(B)** Schematic diagram showing USG transducer probe localizing the site of pseudoaneurysm. **(C)** Schematic diagram showing obliteration of tract following USG guided compression. USG, ultrasound.

**FIG. 4.**
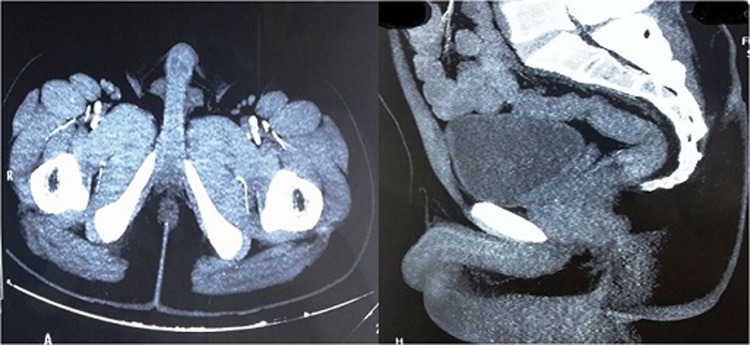
CT angiography images (axial, sagital cuts) showing resolution of pseudoaneurysm after USG-guided compression technique.

A 40-year-old man presented with straining at micturition and progressive thinning of urinary stream for the past 1 year. The patient had a history of urethral stricture disease and underwent OIU 5 years ago. Retrograde urethrogram revealed a short segment mid-bulbar urethral stricture (Fig. 1). The patient underwent uneventful day care OIU performed under spinal anesthesia. OIU was performed with cold cutting knife at 12 o'clock position and 18F silicon catheter placed. Following catheter removal after 7 days, the patient had profuse bleeding per urethra. The patient was admitted in emergency services and worked up. All hematological and coagulation profiles were normal. Bleeding stopped after perineal compression. 18F Foley catheter was reinserted and patient kept on observation for 6 hours during which period there was no fresh bleeding episode. Catheter was again removed after 48 hours following which the patient had repeat profuse urethral bleed. CT angiography was carried out in view of recurrent profuse bleeding, which showed right bulbourethral artery pseudoaneurysm of dimensions 9 × 8 × 8 mm (Fig. 2). The patient was transfused 3 U of packed red blood cells as hemoglobin dropped from 10.4 to 6.2 g/dL. As angioembolization facility was unavailable due to technical reasons, we sought for USG-guided compression repair of urethral artery pseudoaneurysm. Pseudoaneurysm was localized with colour Doppler, and perineal focused USG compression was performed for about 25 minutes (Fig. 3). The patient was kept on observation for 48 hours in hospital; did not have any fresh bleeding episode. On close follow-up, the patient had no recurrence of bleed. Per urethral catheter was removed after 2 weeks and the patient was voiding well. A repeat CT angiography showed resolution of pseudoaneurysm (Fig. 4).

## Discussion

Urethral bleeding can occur due to direct trauma, or after urethral instrumentation, catheterization, OIU, and metal/balloon dilatation. The reason cited for bleeding following OIU is deep cut through the scar tissue into corpus spongiosum and at times into corpora cavernosa. In majority of cases, bleeding is minor and subsides spontaneously or with external compression. Rarely, the bleeding is severe enough to warrant blood transfusion and angioembolization. In the index case, the patient had profuse bleeding per urethra after catheter removal, was admitted in emergency services, and required blood transfusion. Pseudoaneurysm of penile arteries has been described in the literature as one of the causes for urethral bleed following OIU. Bleeding from a pseudoaneurysm secondary to a traumatic urethral catheterization has also been reported. Chiou et al. showed using color Doppler that number and site of urethral arteries vary among individuals in urethral stricture disease. Location of urethral arteries in bulbar urethra was found to be at the 1 to 2 o'clock position in 14% patients, 3 to 4 o'clock in 22%, 5 to 6 o'clock in 17%, 7 to 8 o'clock in 18%, 9 to 10 o'clock in 18%, and 11 to 12 o'clock in 11% patients in his study. Moreover, these arteries may be close to urethral lumen especially in patients who have undergone prior urethral procedure. It is also hypothesized that stricture itself and prior surgery may lead to asymmteric location of urethral arteries.^1^ In the index case, OIU was performed with cold cutting knife and scar incised at 12 o'clock position. The procedure was uneventful and the patient was discharged the same day. Urethral bleed is usually manageable with perineal compression and seldom requires invasive procedure. Infrequently, patient may need invasive procedure like percutaneous embolization of the offending vessel in refractory urethral bleed using thrombotic agents. Transcatheter embolization of the internal pudendal artery has been used to control high-flow priapism, urethrorrhagia after OIU, bleeding from an internal pudendal pseudoaneurysm complicating an ischial pressure sore, and following high-velocity pelvic trauma. Dhabalia et al. reported a case of refractory urethral bleeding following OIU, which was subsequently managed with selective angioembolization of bulbourethral artery.^2^ However, transcatheter angioembolization is not without complications, not to mention, it is technically demanding procedure and requires lots of expertize. Some of the known complication include puncture site pain, bleeding, hematoma formation, coil migration, arterial dissection, chronic pelvic pain, impotence, ischemic urethral stricture, and contrast reactions. USG localization and compression is described procedure for hemostasis. Fellmeth et al. first reported this unique technique of USG-guided compression repair back in 1991 for femoral artery pseudoaneurysm in 27 of 29 patients and suggested safe and technically simple and promising as cost-effective first-line treatment for uncomplicated catheterization-related femoral artery injuries.^3^ Later, Mabjeesh et al. reported perineal USG-guided compression as a noninvasive alternative treatment of high-flow priapism.^4^ The success of this procedure depends on the thrombogenicity of the wall of the track from the artery through the soft tissues. Compression temporarily eliminates the blood flow that inhibits thrombosis, facilitating the formation of a hemostatic plug, and thus converting the pseudoaneurysm into a simple hematoma. USG-guided compression can be justified in all patients without contraindications because it is cost-effective and morbidity is very low. We report a management of USG-guided focused compression technique for bulbourethral artery pseudoaneurysm following OIU. Although successful, it requires monitoring and close follow-up, and if conservative management fails, patient should be considered for early angioembolization.

## Conclusion

Minor bleed is common after OIU. Although the position of urethral arteries and the site of OIU incision are debatable, limiting the incision to avoid going deep into spongiosum especially in previously operated patient may reduce bleeding risk. Perineal USG-guided focused compression may be tried as alternative for refractory urethral bleed before going for invasive angioembolization.

## Abbreviations Used

CT: computed tomography
OIU: optical internal urethrotomy
USG: ultrasound
